# Antinociceptive and anti-inflammatory effect of a standardized fraction of *Oenothera rosea* L'Hér. ex Aiton and its possible mechanism of action in mice

**DOI:** 10.22038/AJP.2022.19616

**Published:** 2022

**Authors:** Rodrigo Vargas-Ruiz, José Eduardo Roa-Coria, Arturo Mora-Olivo, Alejandro Zamilpa, Maribel Lucila Herrera-Ruiz, Rosa Issel Acosta-González, Rosa Mariana Montiel-Ruiz

**Affiliations:** 1 *Center of Biomedical Research of the South, Mexican Institute of Social Security, Morelos, México*; 2 *Section of Postgraduate Studies and Research, Superior School of Medicine, National Polytechnic Institute, Mexico City, México*; 3 *Reynosa-Aztlan Multidisciplinary Academic Unit, Autonomous University of Tamaulipas, Tamaulipas, México*; 4 *Institute of Applied Ecology, Autonomous University of Tamaulipas, Tamaulipas, México*

**Keywords:** Oenothera rosea, Nociception, Inflammation, GABA, Nitric oxide, cGMP

## Abstract

**Objective:**

This study was conducted to investigate the antinociceptive and anti-inflammatory effect of ethyl acetate fraction of *Oenothera rosea* (EAOr) and the mechanism involved, in mice.

**Materials and Methods:**

The antinociceptive activity was tested using chemical- and heat-induced nociception models. The anti-inflammatory activity was tested using carrageenan-induced edema and inflammatory cytokines were measured.

**Results:**

EAOr reduced the licking time on the second phase of the formalin test (100 and 177 mg/kg). The antinociception of EAOr was prevented by L-NAME (10 mg/kg), 1H-[1, 2, 4]-oxadiazolo [4, 3-a]-quinoxalin-1-one (ODQ, 0.1 mg/kg), glibenclamide (10 mg/kg) and bicuculline (1 mg/kg), but not by naloxone (2 mg/kg). Also, EAOr decreased licking time in capsaicin induced-nociception. EAOr did not have effect on withdrawal latency in tail-flick test. Carrageenan-induced paw edema was reduced by EAOr, and TNF-α and IL-1β levels were reduced in mice treated with EAOr by 72.2 and 32.8%, respectively. Furthermore, EAOr did not present side effects as sedation nor gastric injury. Chemical analysis of this fraction showed the presence of glycosylated quercetin derivatives such as quercetin glucoside and quercetin rhamnoside in a 2.5% concentration.

**Conclusion:**

This study demonstrates antinociceptive and anti-inflammatory effect of an organic fraction of *O. rosea* and its possible interaction with the NO-cGMP-K+ channels and GABAergic system and thus, it could be considered a therapeutic alternative.

## Introduction

Pain and inflammation are the most referred problems to seek medical assistance and represent a major healthcare problem. The International Association for the Study of Pain (IASP) defined pain as an unpleasant sensory and emotional experience associated with or resembling that associated with, actual or potential tissue damage (Raja et al., 2020 ▶). In addition, the inflammatory process is the response triggered by a noxious stimulus, and it is considered to be the basic mechanism for tissue reparation (Medzhitov, 2008 ▶). Corticosteroids, non-steroidal anti-inflammatory drugs (NSAIDs), and opioids are important therapeutic agents for inflammation and pain relief (Barnes, 2006 ▶; Schug et al., 2016 ▶). However, most medical treatments are associated with side effects such as gastrointestinal disorders, nephrotoxicity, sedation, and respiratory depression (Shah and Mehta, 2012 ▶). Hence, there is a constant need to search for new molecules to treat several diseases. Lately, natural products have been successfully investigated in order to treat pain and inflammatory diseases (Newman and Cragg, 2020 ▶).

*Oenothera rosea* L'Hér. ex Aiton (Onagraceae) is a native herb from North America, widely distributed among the Mexican territory and commonly known as "Hierba del golpe" (Rzedowski, 2005 ▶; Andrade-Cetto, 2009 ▶). In Mexican folk medicine, aerial parts of this plant are mainly used for pain relief and to decrease inflammation caused by traumatism or tissue wounds, and it is also used to treat stomach illness and headache (Argueta et al., 1994 ▶; Singh et al., 2012 ▶). Previous studies on aqueous extracts of aerial parts from *O. rosea* showed an anti-inflammatory effect on carrageenan-induced paw edema (Meckes et al., 2004 ▶) and cotton pellet induce granuloma formation (Márquez-Flores et al., 2009 ▶). Also, fibrinolytic and antiplatelet activity of this species was determined in *in vitro* and *in vivo* models, respectively (Díaz Porras et al., 2011 ▶). The analgesic effect of this species has been described using acetic acid-induced nociception (Márquez-Flores et al., 2018 ▶). Additionally, a previous study has demonstrated that phenolic compounds are mainly distributed among this species, especially quercetin glycosides (Vargas-Ruiz et al., 2020 ▶). Also, there is evidence about the presence of coumarins, tannins, alkaloids and saponins in this species (Márquez-Flores et al., 2009 ▶; Díaz Porras et al., 2011 ▶). 

Based on the traditional uses, researchers provide scientific information revealing the anti-inflammatory effect and antinociceptive effect of this species. However, there is still a lack of evidence of the mechanism through which the antinociceptive effect is produced and it is necessary to investigate other bioactive chemical compounds present in the extract. Thus, the aim of this study was to assess the antinociceptive and anti-inflammatory effects of *O. rosea* and to identify the signaling pathway through which the antinociceptive effect is produced. Also, the possible side effects associated with analgesic drugs such as motor activity and gastric disorders were investigated.

## Materials and Methods

**Plant material**

*Oenothera rosea *was collected in Ciudad Victoria, Tamaulipas and was identified by Dr. Arturo Mora-Olivo from the Instituto de Ecologia Aplicada, Universidad Autonoma de Tamaulipas. A plant specimen was deposited as reference at the herbarium UAT and was assigned the voucher number 03358.

**Extract preparation**

The aerial parts of *O. rosea *were washed and dried at room temperature (25±3ºC) for three days. The dried material was pulverized (200 g) and extracted by maceration in a hydroalcoholic solution (70% distilled water: 30% ethanol) for 48 h with intermittent agitation. The liquid extract was filtered, and the solvent was eliminated by reduced pressure distillation in a rotary evaporator at 55°C. The resulting semisolid extract was suspended in 200 ml of distilled water and partitioned by liquid-liquid fractionation using ethyl acetate (200 ml, 3 times). The organic phase was filtered and dried by a rotary evaporator and stored at 4°C, to obtain the ethyl acetate fraction (EAOr). 

**Standardization of EAOr fraction**

We have previously described two quercetin glycosides to be the major constituents of this fraction, so a quantitative analysis of these flavonoids has been done using chromatographic analysis in a Waters 2695 separation module system equipped with a Waters 996 photodiode array detector and Empower Pro software (Waters Corporation, USA) (Vargas-Ruiz et al., 2020 ▶). Chemical separation was achieved using a Supelcosil LC-F column (4.6 mm × 250 mm i.d., 5 μm particle size) (Sigma-Aldrich, Bellefonte, PA, USA). The mobile phase consisted of a 0.5% trifluoroacetic acid aqueous solution (solvent A) and acetonitrile (solvent B). The gradient system was as follows: 0–1 min, 0% B; 2–3 min, 5% B; 4–20 min, 30% B; 21–23 min, 50% B; 24–25 min, 80% B; 26–27 100% B and 28–30 min, 0% B. The flow rate was maintained at 0.9 ml/min and the sample injection volume was 10 μl of sample diluted in methanol. Both, commercial standards of quercetin glucoside (SIGMA, 0000, City) and quercetin rhamnoside (SIGMA, 0000, City) were used as references for the detection and quantification of flavonoids in the *O. rosea* treatment. The amount of these constituents was estimated by interpolation of peak areas and comparison with calibration curves developed with commercial standards of each compound. Both calibration curves were linear in the range of 6.25–100 μg quercetin glycoside/ml, and sample concentration was calculated using linear regression

**Animals**

Experiments were performed on 204 male ICR mice (28-32 g), obtained from the Universidad Autonoma del Estado de Morelos. All experiments followed the Guidelines on Ethical Standards for Investigation of Experimental Pain in Animals (Zimmermann, 1983 ▶) and Mexican Official Norm for Animal Care and Handing (NOM-062-ZOO-1999). Efforts were made to minimize animal suffering and to reduce the number of animals used. Mice were used only once, and they were housed in a climate-controlled room with a 12 hr light/dark cycle. Twelve hours before experiments, mice were divided into groups of six, food was withheld, but animals had free access to water. At the end of the experiment, animals were sacrificed in a CO_2_ chamber. The EAOr fraction was suspended in a vehicle (tween 20, 5% in saline solution) and then, administered orally (p.o.) in a single dose, in a volume of 0.1 ml/10 g body weight (BW). The extract was administered to the mice 30 min before the experiments. 

**Drugs**

Indomethacin, capsaicin, tween 20, dexamethasone, and lambda-carrageenan, N(G)-Nitro-L-arginine methyl ester (L-NAME), 1H-[1, 2, 4]-oxadiazolo [4, 3-a]-quinoxalin-1-one (ODQ), glibenclamide, bicuculline and naloxone were obtained from Sigma Chemical Co, SO, USA. Tramadol was purchased from Grünenthal SA de CV, Mexico. Formaldehyde was obtained from JT Baker, PA, USA. Solvents were analytical grade. 

**Measurement of antinociceptive activity**

**Formalin test**

The test begins with placing each mouse in an open Plexiglass observation cylinder (20 cm in diameter) with mirrors to allow them to get used to the environment. Twenty minutes later, each animal was removed and 20 µl of diluted formalin (2%) was administered subcutaneously (s.c.) into the right hind paw of the mouse with a 30-gauge needle. The animals were divided into six groups and pre-treated with EAOr (30, 56, 100 and 177 mg/kg, p.o.), indomethacin (10 mg/kg, i.p.) or vehicle (Tween 20, 5%), 30 min before formalin injection, and returned to the chamber for observation. The time of licking of the injected paw was defined as a nociceptive response, which was recorded during a 40 min period, accordingly to the literature, the first phase and second phase of the formalin test is produced in 0-10 min and 15-40 min, respectively, after formalin injection (Hunskaar and Hole, 1987 ▶).

**Capsaicin test**

The capsaicin test was performed as previously described by Sakurada et al. (1992) ▶. Similar observation chamber and acclimatization to the environment were used in this test as described above. Nociceptive behavior was induced by the administration of capsaicin (1.6 µg/20 µl, s.c.) into the right hind paw. The total time of licking of the injected paw was recorded for 5 min after the capsaicin injection. Animals were divided into three groups and received a single dose of vehicle (tween 20, 5%), indomethacin (10 mg/kg, i.p.), or EAOr (100 mg/kg, p.o.), 30 min before capsaicin injection.

**Tail flick test**

The tail ﬂick test was carried out according to the method described by D’Amour and Smith (1941) ▶. Three groups of mice were treated with vehicle (Tween 20, 5%), tramadol (50 mg/kg, i.p.), and EAOr (100 mg/kg, p.o.). The distal half of each mice tail was positioned on the source of radiant heat emitted by the analgesia meter (IITC Life Science Inc., model 33). The reaction latency time (in seconds) was measured as the time from the onset of the heat exposure to the withdrawal of the tail. The cut-off point was considered 10 sec to avoid tissue damage. The latency times were determined in 0 min (basal time), 30, 60, 90, and 120 min from the time of treatment. 

**Antinociceptive mechanism of action of the EAOr in the formalin test**

To evaluate the involvement of different pathways in nociception, different antagonists of pain pathways were administered to animals. Fifteen minutes before EAOr (100 mg/kg, p.o.) or vehicle administration, animals were treated (subcutaneously) with 10 mg/kg of L-NAME (a nitric oxide (NO) synthase inhibitor), 0.1 mg/kg of ODQ (an inhibitor of the guanylate cyclase), 1 mg/kg of glibenclamide (an ATP-sensitive K+-channels blocker), 2 mg/kg of bicuculline (a GABA_A_ receptor antagonist), or 2 mg/kg of naloxone (an opioid receptor antagonist). Then, 30 min after EAOr or vehicle administration, each animal received an intraplantar injection of 20 µl of 2% formalin to register a nociceptive behavior as described above. 

**Measurement of anti-inflammatory activity**

**Carrageenan-induced paw edema**

In order to evaluate the anti-inflammatory activity of EAOr, carrageenan-induced paw edema method was used as previously described, in mice (Levy, 1969 ▶). Briefly, inflammation was induced in the intra-plantar tissue of the right hind paw of mice by subcutaneous injection of 20 μl of 1% lambda carrageenan. The mice were pretreated with vehicle (Tween 20, 5%), dexamethasone (1 mg/kg, p.o.), and EAOr (100 mg/kg, p.o.) 30 min before administration of carrageenan. The inflammation was measured immediately after the dose administration (basal) and then 0.5, 1, 2, 4, and 24 hr after carrageenan injection using a digital plethysmometer (Ugo Basile, 37140). The increase in the paw volume was calculated by subtracting the initial paw volume from the paw volume measured at each time point.

**Inflammatory mediators**

Tissue levels of the proinflammatory cytokines interleukin (IL)-1β and tumor necrosis factor (TNF)-α in the lumbar spinal cord were determined with commercial ELISA kits from Cayman Chemical (Item No 583311) and USBiologicals Life science (Item No 144066), respectively. Tissue was collected before administration of treatments (basal measurement, naive group) and 4 hr after intraplantar carrageenan injection in the right paw (experimental groups). Groups of mice were pre-treated as described in the section of carrageenan-induced paw edema.

**Ethanol- evoked gastric injury**

Animals were divided into three groups: Vehicle (Tween 20, 5%, p.o.), omeprazole (20 mg/kg, p.o.), and EAOr (100 mg/kg, p.o.). Intragastric administration of ethanol (0.1 ml/10 g) evoked gastric injury. Thirty minutes after administration of treatments, animals received intragastric ethanol. Animals were sacrificed 1 h after in a CO_2_ chamber and stomachs were rapidly removed and processed by ImageJ (software of National Institute of Health, USA) to quantify the ulcer area produced (Reyes-Garcia et al., 2007 ▶). 

**Rotarod test**

Animals were divided into two groups: Vehicle (Tween 20 5%, p.o.) and EAOr (100 mg/kg, p.o.). Mice were trained 24 h before the test, to familiarize to use the rotarod system (UgoBasile 47650, Italy) at 15 rpm for at least 180 sec. After oral administration of treatments, motor coordination was measured at 0.5, 1, 1.5, 2, 4, and 24 hr (Dunham and Miya, 1957 ▶).

**Statistical analysis**

The data are expressed as the means±standard error of the mean (S.E.M.). One-way analysis of variance (ANOVA) followed by Dunnett’s *post-hoc* test was used for formalin, capsaicin, and gastric damage test. Meanwhile, two-way ANOVA followed by Dunnett´s *post-hoc* test was used for the tail-flick test and carrageenan-induced edema test. Values were considered significant when p<0.05. 

## Results

**Standardization of EAOr fraction**

**Figure 1:**
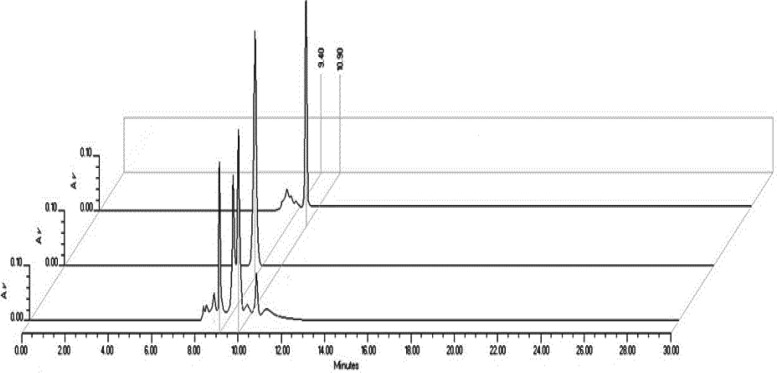
Chromatographic comparison of HPLC analysis of EAOr and quercetin glycosides analytical reference standard; Quercetin glucoside standard (1) and quercetin rhamnoside (2), all fingerprints were extracted at λ=350 nm

The organic fraction was subjected to an HPLC analysis to detect and quantify major constituents of the mixture with antinociceptive and anti-inflammatory effects. HPLC analysis showed the presence of two major constituents present in EAOr (R_t_ between 9.4-10.9 min); quercetin glucoside and quercetin rhamnoside (peak 1 and 2, respectively) were identified by direct comparison with commercial standards (Figure 1). After determining quercetin derivatives to be the major constituents, HPLC quantification of these compounds was carried out. After data analysis, quercetin glucoside and quercetin rhamnoside concentrations were calculated 11.25 and 13.8 g/mg EAOr, respectively. Thus, EAOr is a fraction containing 1.12% of quercetin glucoside and 1.38% of quercetin rhamnoside, with a total of 2.5% of quercetin glycosides. Since EAOr at 100 mg/kg produced the highest biological activity, every administration of this dose was equivalent to 11.25 g/10 g BW and 13.8 g/10 g BW of quercetin glucoside and quercetin rhamnoside, respectively.

**Antinociceptive effect of** ***O. rosea*** **on acute nociception in mice**

**Figure 2:**
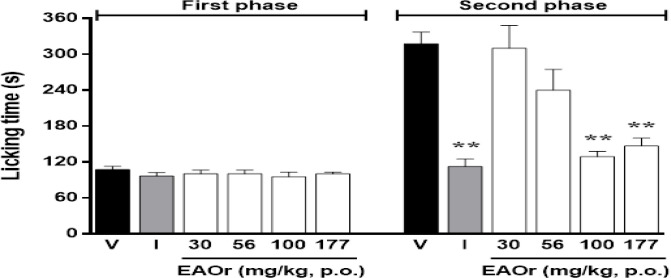
Effects of EAOr on the first and second phase on formalin-induced nociception. Mice were pretreated with vehicle (Tween 20, 5%, p.o.), indomethacin (10 mg/kg, i.p.) or EAOr (30-177 mg/kg, p.o.) 30 min prior to formalin solution. Each column represents the mean±S.E.M. of 6 animals. The asterisks denote the significance level **p<0.01 compared to the control group (vehicle) as assessed by ANOVA followed by Dunnett’s *post-hoc* test

In order to assess the antinociceptive effect of the EAOr fraction, pain behavior was evaluated in three acute pain models. In the formalin test, the licking time was not significantly different among EAOr treatments (30-177 mg/kg, p.o.) in phase 1, only doses of 56,100 and 177 mg/kg significantly decreased (p<0.01) licking time in phase 2 (Figure 2). The maximum inhibition percentage of nociceptive behavior caused by EAOr at 100 mg/kg was 53.11%. Positive control indomethacin at 10 mg/kg (i.p.) also significatively decreased licking time only in phase 2 (p<0.01).

**Antinociceptive mechanism of action of the EAOr in the formalin test**

**Figure 3:**
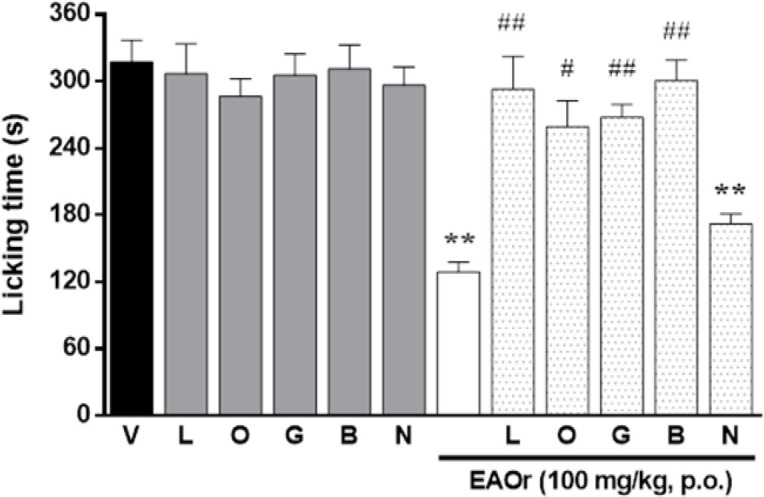
Effect of L-NAME (L,10 mg/kg, s.c), ODQ (O, 0.1 mg/kg, s.c.), glibenclamide (G, 10 mg/kg, s.c.), bicuculline (B, 2 mg/kg, s.c.) and naloxone (N, 2 mg/kg, s.c.) on the antinociceptive effect of EAOr (100 mg/kg, p.o.) in the second phase of formalin-induced nociception. Each column represents the mean±S.E.M. for 6 animals. The asterisks denote the significance level *p<0.05 and **p<0.01 compared to the control group (vehicle) and #p<0.05 and ##p<0.01 compared to EAOr group as assessed by ANOVA followed by Dunnett’s *post-hoc* test

The subcutaneous administration of a nitric oxide synthase inhibitor (L-NAME, 10 mg/kg), or an inhibitor of the guanylate cyclase (ODQ, 0.1 mg/kg), or an ATP-sensitive K^+^-channels blocker (glibenclamide, 1 mg/kg), significantly inhibited the antinociceptive effect induced by EAOr (100 mg/kg, p.o.) in the second phase of formalin-induced nociception (Figure 3). Moreover, the subcutaneous injection of a GABA_A_ receptors antagonist (bicuculline, 2 mg/kg), significantly reduced the antinociceptive effect of EAOr (100 mg/kg, p.o.) in the second phase of formalin-induced nociception. On the other hand, naloxone (2 mg/kg), a non-selective antagonist of the opioid receptors, did not modify the antinociceptive effect of EAOr (100 mg/kg, p.o).

**Figure 4:**
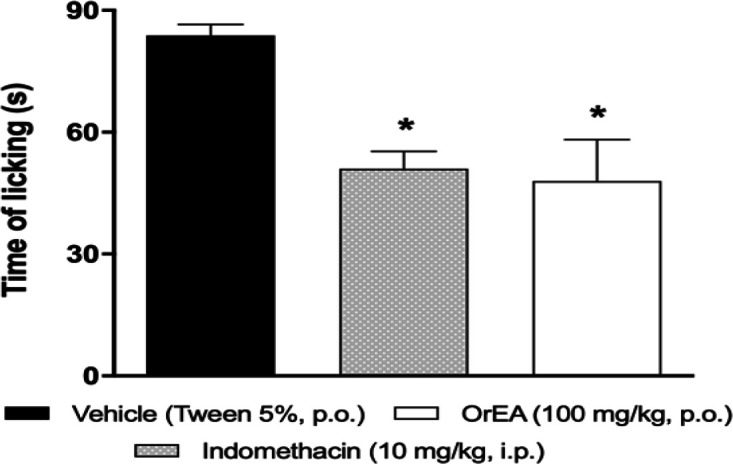
Effects of EAOr on capsaicin test in mice. Mice were pretreated with vehicle (Tween 20, 5%, p.o.), indomethacin (10 mg/kg, i.p.) or EAOr (100 mg/kg, p.o.) 30 min prior to nociceptive stimuli. Each column represents the mean±S.E.M. for 6 animals. The asterisks denote the significance level *p<0.05 compared to the control group (vehicle) as assessed by ANOVA followed by Dunnett’s *post-hoc* test

The results obtained in the capsaicin test are shown in Figure 3. At dose given orally of 100 mg/kg, the dose with the maximum antinociceptive effect in the formalin test, EAOr fraction produced a significant inhibition in the licking response of 42.72%, whereas indomethacin reduced to 39.14% (Figure 4). For thermal nociception, the tail-flick test was performed. The dose of 100 mg/kg (p.o.) of EAOr did not produce a significative difference compared to the control group (Tween 20, 5%, p.o.), whereas tramadol (50 mg/kg, i.p.) caused a significant increase in the reaction time to thermal stimuli as compared to the control group (Table 1).

**Anti-inflammatory effect of** ***O. rosea*** **on acute edema in mice**

**Figure 5:**
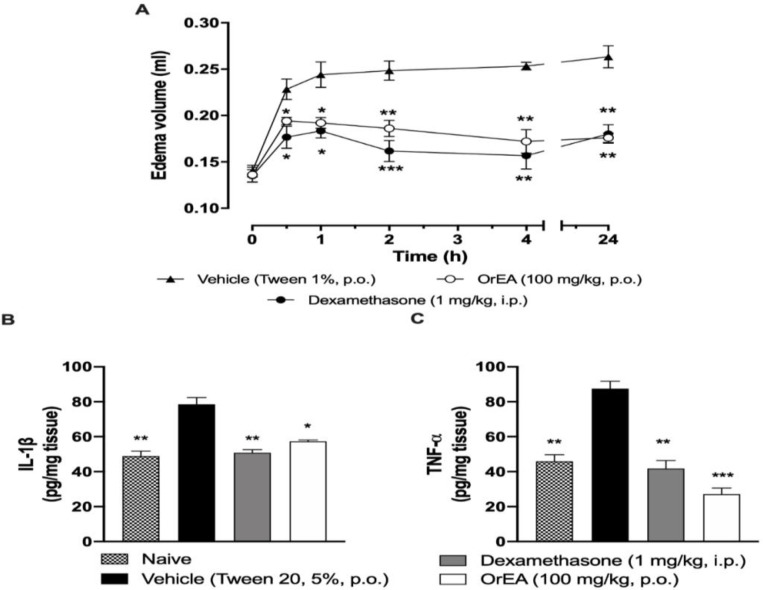
Anti-inflammatory effect of EAOr on paw edema formation (panel A), IL-1β (panel B) and TNF-α (panel C) levels in carrageenan-induced edema. Mice were pretreated with vehicle (Tween 20, 5%, p.o.), dexamethasone (1 mg/kg, i.p.) or EAOr fraction (100 mg/kg, p.o.). Each column/point represents the mean±S.E.M. for 6 animals. *p<0.05, **p<0.01 and ***p<0.001 Statistically significant difference compared to the control group (vehicle) as assessed by one-way ANOVA followed by Dunnett´s *post-hoc* test

In Figure 5, the anti-inflammatory effect of EAOr fraction (100 mg/kg, p.o.) is presented. In the carrageenan-induced paw edema, a significant reduction (p<0.01) of the edema was detected in the early phase (2 hr) and the anti-inflammatory activity was persistent until 24 hr after the administration. TNF-α and IL-1β levels were evaluated on the lumbar spinal cord followed by the stimulation of carrageenan injection. Both cytokines were increased 4 hr after intraplantar carrageenan injection, EAOr fraction (100 mg/kg, p.o.) was able to significantly diminish the concentration of both TNF-α and IL-1β by 72.2 and 32.8%, respectively, with respect to the control group. Meanwhile, dexamethasone reduced TNF-α and IL-1β concentration by 49.91 and 36.30%, respectively (Figure 4B and Figure 4C).

**Table 1:** Effect of EAOr on the tail-flick test. Mice received vehicle (Tween 20, 5%, p.o.), tramadol (50 mg/kg, i.p.), or EAOr (100 mg/kg, p.o.) 30 min before to thermal stimuli

Treatment	Doses	Latency (h)				
		0	0.5	1	1.5	2
Vehicle	0.1 ml/10 gr	2.73±0.28	2.78±0.27	2.78±0.13	3.14±0.11	2.98±0.24
Tramadol	50 mg/kg	2.74±0.22	8.75±0.75*	9.32±0.42*	7.85±0.92*	8.09±1.17*
EAOr	100 mg/kg	2.70±0.18	3.29±0.23	3.92±0.43	3.14±0.24	3.21±0.24

Each point represents the mean±S.E.M. for 6 animals. *Statistically significant difference (p<0.05) compared to the control group (vehicle), as assessed by two-way ANOVA followed by Dunnett´s *post-hoc* test**.**

**Gastric protection and motor activity evaluation**

**Figure 6:**
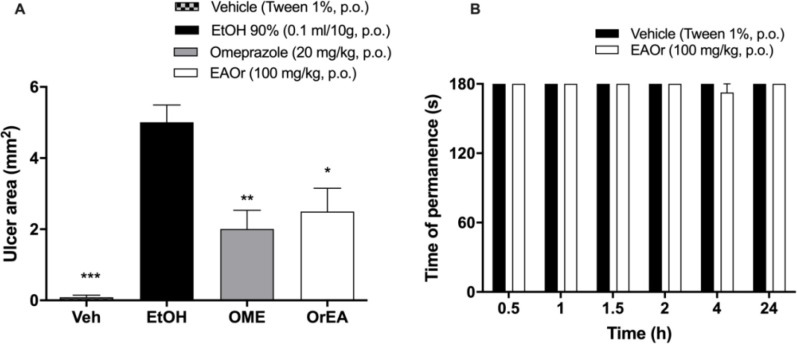
Gastric protection on ethanol-induced gastric ulcer (Panel A) and motor evaluation on rotarod test (Panel B) of EAOr. *p<0.05, **p<0.01 and, ***p<0.001 Statistically significant difference compared to the control group (vehicle), as assessed by one-way ANOVA followed by Dunnett´s test. Veh: vehicle, Veh: EAOr without ethanol administration, EtOH: damage group, OME: Omeprazole, EAOr: EAOr with ethanol administration

EAOr was tested in the ethanol-induced gastric injury and the rotarod test for commonly known side effects produced by NSAIDs and opioids such as gastric damage or sedation, respectively. In Figure 6-A, results show a reduction in the ulcer triggered by ethanol on omeprazole (20 mg/kg, p.o.) and EAOr (100 mg/kg, p.o.) treated groups by 59.9 and 50.1%, respectively. On the other hand, as shown in Figure 6-B, oral administration of EAOr showed no modification in the motor activity on the rotarod test.

## Discussion

*Oenothera rosea* is a medicinal plant used in the folk medicine of the north and center of Mexico. According to previous phytochemical reports, there are several compounds present in the aerial parts of *O. rosea* including flavonoids, coumarins, saponins, and tannins (Márquez-Flores et al., 2009 ▶; Díaz Porras et al., 2011 ▶; Almora-Pinedo et al., 2017 ▶). We previously described the presence of two quercetin glycosides as the major compounds in this organic fraction of *O. rosea*, also, gallic acid, myricetin 3-O-glucoside, and tamarixetin 3-O-rutinoside as minor compounds (Vargas-Ruiz et al., 2020 ▶). Plants with a high quantity of flavonoids can induce anti-nociceptive and anti-inflammatory activities *in vivo* and *in vitro* (Guardia et al., 2001 ▶; Pinho-Ribeiro et al., 2015 ▶). 

The formalin test is a method widely used to study acute inflammatory pain and drugs with antinociceptive potential. This model produces a biphasic nociceptive response to formalin injection in the paw (Tjolsen et al., 1992 ▶). The first phase is associated with direct effect of formalin on activation of peripheral afferent C-fibers, while the second phase involves inflammatory processes by the release of serotonin, histamine, bradykinin, and prostaglandins (PG) from injured tissue (Hunskaar and Hole, 1987 ▶; Bannon and Malmberg, 2007 ▶). Opioids (*e.g. *morphine) inhibit both phases, while NSAIDs (*e.g. *indomethacin) inhibit the second phase only (Noriega et al., 2020 ▶). EAOr fraction inhibited licking behavior in the second phase of formalin test with the highest activity showed at the dose of 100 mg/kg (p.o.), suggesting that its antinociceptive effect is related to inflammatory pain and it could be related to its anti-inflammatory potential. The analgesic effect of *O. rosea *was previously described (Márquez-Flores et al., 2018 ▶), as well as, its major chemical component quercetin (Carullo et al., 2017 ▶), yet, pain pathways involvement in the antinociceptive effect had to be investigated.

Hence, the mechanism involved in the antinociceptive effect of EAOr was investigated. To assess the involvement of the NO-cGMP-K+ channel pathway, pre-administration with L-NAME, ODQ, and glibenclamide was done. NO is a biological messenger that can be associated with pain generation and transmission (Janicki and Jeske-Janicka, 1998 ▶). NO has been evidenced to be involved in pain generation at the spinal and cerebrocortical loci of the somatosensory pathway. It has been demonstrated that NO and inflammatory cytokines are correlated in peripheral pain. Findings suggest a feedback loop between NO and cytokines on inflammatory pain (Cury et al., 2011 ▶). NO production is accompanied by an increase in cGMP, with a consequent stimulation of protein kinase G (PKG) (Francis et al., 2010 ▶). Increased cGMP and PKG in peripheral sensory neurons stimulate K+ channels opening, leading to repolarization and inhibition of the action potential generation (Ferreira et al., 1991 ▶; Sachs et al., 2004 ▶).

Experimental data showed that L-NAME prevented the antinociceptive effect of EAOr, suggesting that EAOr effect could be associated with NO production. Additionally, the antinociceptive effect of EAOr was attenuated by pre-administration of ODQ and glibenclamide, being related to cGMP and K+ channels. Thus, hyperpolarization of the membrane caused by opening of potassium channels is suggested to be a key event for the antinociceptive effect of EAOr. 

Additionally, GABAergic receptors signaling pathway was investigated using bicuculline, a competitive antagonist of the GABA_A_ receptors. The GABA is an important inhibitory neurotransmitter of the central nervous system (CNS) in mammals, acting at inhibitory synapses in the brain by binding with specific receptors in the pre-and postsynaptic plasma membranes (Xu et al., 2008 ▶). In normal conditions, GABAergic interneurons act as a gatekeeper controlling nociceptive signals from the periphery to the spinal cord towards higher CNS (Zeilhofer et al., 2009 ▶). Our results showed that pre-administration of bicuculline prevented the antinociceptive effect of EAOr, suggesting that its antinociceptive effect could be related to GABAergic receptors. 

On the other hand, opioid receptor involvement in EAOr antinociceptive effect was studied for central acting analgesia, using naloxone. Naloxone is a nonselective opioid receptors antagonist (Rzasa Lynn and Galinkin, 2018). Since the antinociceptive effect of EAOr was not prevented by pre-administration of naloxone, opioid receptors inhibition mediated analgesia was discarded. In order to confirm the absence of spinal analgesia participation in the effect of EAOr, the tail-flick test was used. This test is widely used to evaluate the antinociceptive effect at the spinal level (Hole and Tjølsen, 2007 ▶). EAOr fraction did not produce antinociception in this model, suggesting that EAOr fraction is not a mixture of substances with activity at spinal level. 

Lastly, transient receptor potential vanilloid (TRPV) mediated antinociception of EAOr was analyzed using the capsaicin-induced nociception. Capsaicin is the main active ingredient of chili peppers; it produces pain by selectively activating polymodal nociceptive neurons. This substance is a selective agonist of the TRPV1, described on C-type nociceptive neurons (Frias and Merighi, 2016 ▶). This test is useful to investigate the physiologic responses, including chemogenic nociception and neurogenic inflammation evoked by activation of C-fibers. The peripheral injection of capsaicin releases neuropeptides, excitatory amino acids (glutamate and aspartate), nitric oxide, and pro-inflammatory mediators in the periphery transmitting nociceptive information to the spinal cord (Sakurada et al., 1992 ▶). EAOr fraction significantly reduced the neurogenic nociception produced by subcutaneous capsaicin administration on the paw. Hence, the antinociceptive activity of EAOr fraction might depend in part on the abundant presence of flavonoids, whose mechanism of action may involve antagonism of TRPV1 receptors (Rossato et al., 2011 ▶). All of this is in concordance with the antinociceptive effect of quercetin, which glycosides are the major compounds present in the EAOr fraction, showing its interaction with TRPV1 receptors among other pathways of pain transmission such as interaction NO, serotonin, and GABA systems (Filho et al., 2008 ▶). 

Carrageenan-induced paw edema is a well-established animal model to assess the anti-inflammatory effect of novel compounds. Edema formation due to carrageenan in paw is a biphasic event; the initial phase (0-1.5 h) involves the activation of resident macrophages, mast cells, and endothelial cells, which results in the release of several proinflammatory cytokines and mediators such as TNF-α, IL-1β, IL-6, NO, histamine and serotonin (Ren and Dubner, 2010 ▶). In the second phase (2-24 h) edema has been shown to be the result of overproduction of PGs. Oral administration of EAOr demonstrated that it is effective in the early phase of inflammation, also the anti-inflammatory effect of EAOr remains significant up to 24 h after carrageenan injection, this was in concordance with literature as this species anti-inflammatory effect is widely described (Meckes et al., 2004 ▶; Márquez-Flores et al., 2009 ▶). Even though anti-inflammatory activity was previously described, we are providing evidence about cytokine modulation over carrageenan-induced paw edema. Hence, the anti-inflammatory effect produced by the fraction could be related to cytokine modulation. There is previous evidence of natural products, modulating proinflammatory cytokine production (Park et al., 2008 ▶). Moreover, the interaction of proinflammatory cytokines (IL-1β and TNF-α) has been described in pain modulation (Zhang and An, 2007 ▶); therefore IL-1β and TNF-α decrease also could be related to EAOr antinociceptive effect.

NSAIDs are commonly used to relieve pain and inflammation. However, long-term use of these drugs may produce gastrointestinal ulcers bleeding and renal damage (Hatt et al., 2018 ▶). In this work, we tested EAOr fraction in gastric injury evoked by ethanol, a solvent that produces gastric injury characterized by mucosal edema, cellular exfoliation, and petechial bleeding, through solubilization of mucus constituents in the stomach, and a concomitant fall in the transmucosal potential difference, increases the fluxes of Na^+^ and K^+^ into the lumen, pepsin secretion, the loss of H^+^ ions and the histamine content in the lumen (Liang et al., 2018 ▶). Gastric injury evoked by ethanol could be prevented by cytoprotective agents such as PG’s synthesis promoters (Hiruma-Lima et al., 2001 ▶). Antinociceptive dose of EAOr fraction showed a gastroprotective effect on the ethanol evoked gastric injury. There is a protective effect produced by the major compounds of EAOr, which are known to be natural antioxidant compounds, such as quercetin (Coşkun et al., 2004 ▶). On the other hand, modification of motor performance was tested as a side effect produced by opioids and some adjuvants medication for pain treatment (Hooman Khademi et al., 2016 ▶). Rotarod test has been used to study drugs that alter motor coordination (Mann and Chesselet, 2015 ▶), and we tested EAOr fraction on motor coordination and balance. Mice pretreated with EAOr (100 mg/kg, p.o.) showed no alteration in the behaviors evaluated on the rotarod test.

The ethyl acetate fraction of aerial parts of *O. rosea *was proven as a natural remedy for the treatment of nociception and inflammation. This is the first evidence of the involvement of the NO-cGMP-K+ channels pain pathway and GABAergic system of this species. Also, this fraction produced an inhibition of the production of cytokines in the lumbar spinal cord, which can be partially related to the antinociceptive and anti-inflammatory effect. 

## Conflicts of interest

The authors have declared that there is no conflict of interest.
